# Breakthroughs in deep tumour penetrating nano‐phototheranostics for tumour ablation

**DOI:** 10.1002/ctm2.70188

**Published:** 2025-01-12

**Authors:** Min‐Jun Baek, Sang Min Lee, Dae‐Duk Kim, Jae‐Young Lee

**Affiliations:** ^1^ Gordon Center for Medical Imaging Department of Radiology Massachusetts General Hospital and Harvard Medical School Boston Massachusetts USA; ^2^ College of Pharmacy and Research Institute of Pharmaceutical Sciences Seoul National University Seoul Republic of Korea

## 1 THE CHALLENGES OF PHOTODYNAMIC THERAPY IN ANTICANCER TREATMENTS

Photodynamic therapy (PDT), which leverages reactive oxygen species to eliminate cancer cells, offers a promising alternative to conventional cancer treatments. By utilising light‐activated photosensitizers (PSs), PDT achieves precise tumour targeting while minimising damage to surrounding healthy tissues. This targeted approach positions PDT as a potential replacement for surgery and radiation therapy in selected cases. However, the clinical utility of PDT in managing solid tumours remains constrained by several critical challenges, including suboptimal tumour accumulation and limited penetration of PSs into tumour tissues.^1^ These barriers often lead to incomplete tumour remission, rendering PDT less effective compared to traditional therapies. Addressing these limitations requires innovative PS delivery systems to enhance the performance of PDT.

Nanoparticle (NP)‐based delivery systems have emerged as a promising approach to overcome these obstacles. By leveraging their unique properties, NPs can improve the solubility, stability and tumour selectivity of PSs. However, NP‐based approaches often fail to achieve satisfactory outcomes due to poor penetration of NPs into tumour tissues.^2^ Overcoming these challenges requires innovative tumour‐targeted delivery systems that enhance both the specificity and penetrability of NPs.

## 2 THERAPEUTIC POTENTIAL OF NANO‐PHOTOTHERANOSTICS FOR IMAGE‐GUIDED PHOTOTHERAPY

**FIGURE 1:**
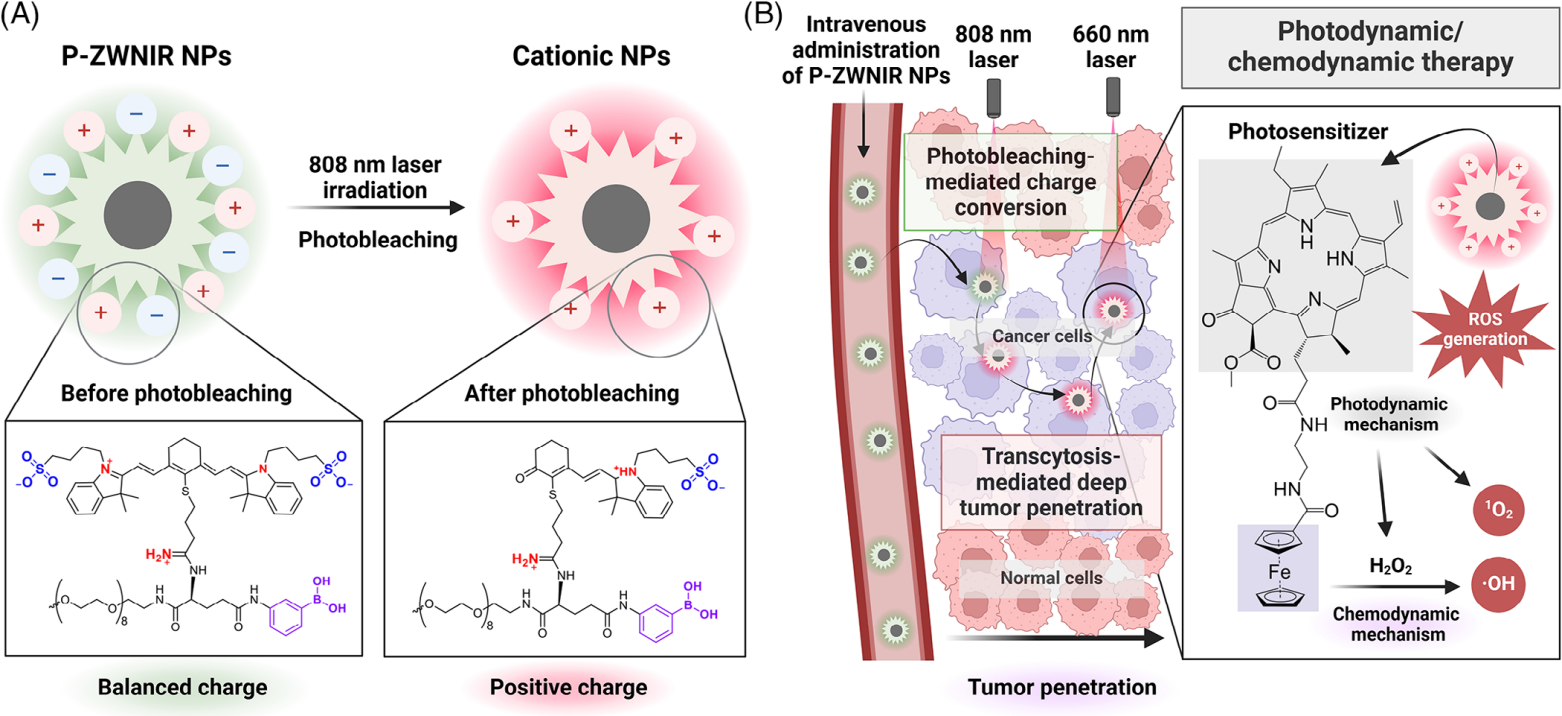
Schematic illustration of charge conversion and tumour penetration mechanism of photobleaching‐mediated charge‐convertible zwitterionic near‐infrared nanoparticles (P‐ZWNIR NPs) (created with Biorender.com).^3^ (A) Photobleaching‐mediated charge conversion process under 808 nm laser irradiation. (B) Transcytosis‐mediated deep tumour penetration and photodynamic/chemodynamic therapy cascade of P‐ZWNIR NPs.

Our recent study introduced photobleaching‐mediated charge‐convertible zwitterionic near‐infrared NPs (P‐ZWNIR NPs), representing a transformative innovation in nano‐phototheranostics.^3^ These multifunctional NPs address critical limitations of PDT and nanotherapeutics by integrating advanced design principles to enhance targeting and penetration in solid tumours. P‐ZWNIR NPs feature a photobleaching‐mediated charge conversion mechanism. Initially, the NPs are designed to have zwitterionic surface charge to ensure colloidal stability, reduce off‐target adsorptions and facilitate tumour‐selective accumulation upon intravenous injection. The outer zwitterionic near‐infrared (NIR) fluorophore component of the NPs undergoes photobleaching upon exposure to an 808 nm laser, which induces charge conversion to a cationic charge (Figure 1A).

A key innovation of P‐ZWNIR NPs is rapid and efficient charge conversion within tumour tissue, which further facilitates deep tumour penetration. Upon exposure to 808 nm laser, the zwitterionic surface transitions to a cationic state via photooxidative cleavage of the NIR fluorophore component in the NPs. The resulting cationic charge facilitates transcytosis of NPs, enabling them to cross multiple layers of cells in tumour tissue. By promoting active penetration, P‐ZWNIR NPs achieved homogeneous distribution of PSs throughout the tumour tissue (Figure 1B).

**FIGURE 2:**
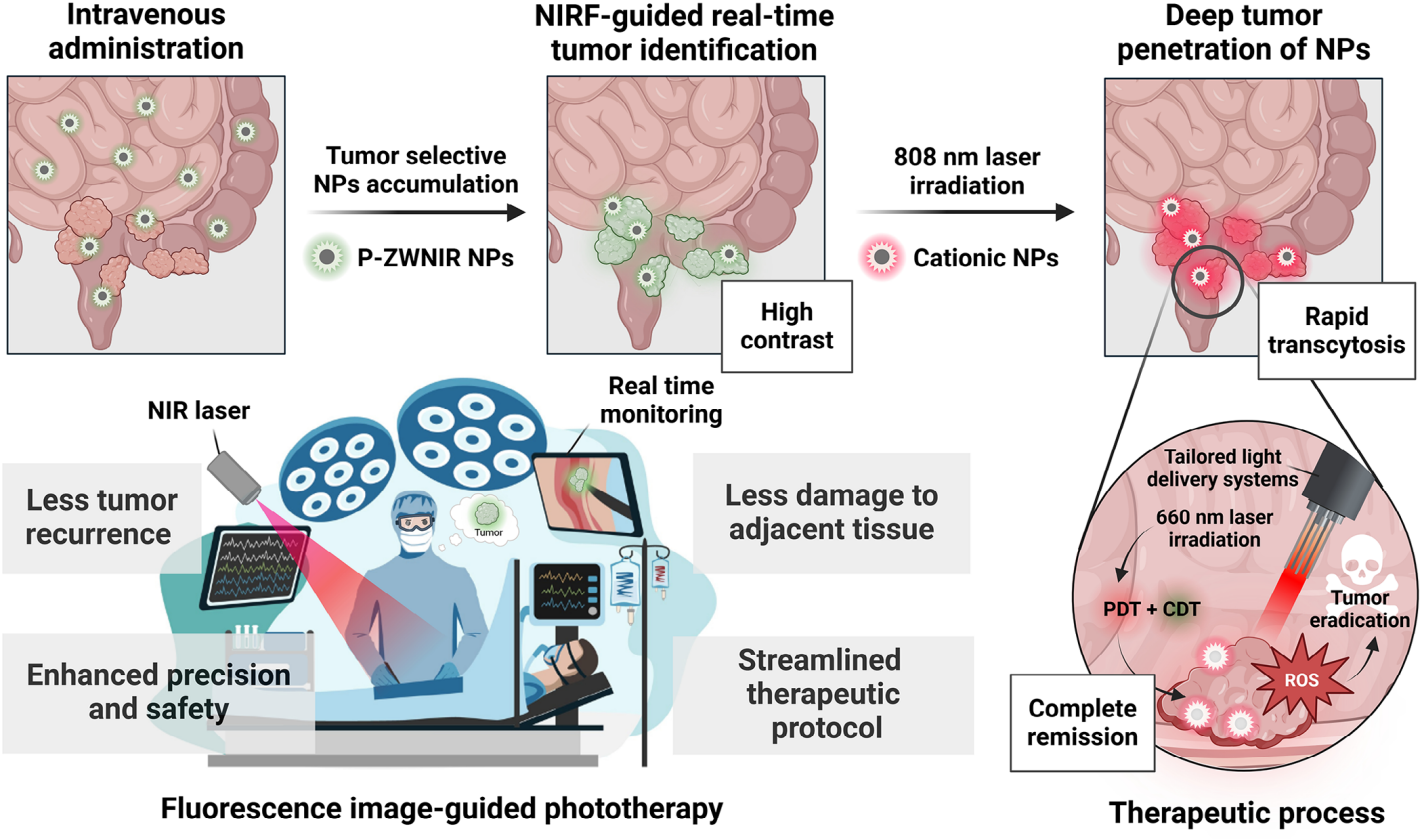
Schematic illustration of near‐infrared fluorescence (NIRF) image‐guided phototherapy utilising photobleaching‐mediated charge‐convertible zwitterionic near‐infrared nanoparticles (P‐ZWNIR NPs) (created with Biorender.com).

In orthotopic rectal tumour‐bearing mouse models, intravenous administration of P‐ZWNIR NPs resulted in a tumour‐to‐background ratio as high as 10 at 8 h post‐injection, enabling real‐time near‐infrared fluorescence (NIRF) tumour imaging for precise localisation. Following localisation, P‐ZWNIR NPs were activated using a sequential two‐step laser irradiation protocol. The first laser (808 nm) triggered charge conversion to promote tumour penetration, while the second laser (660 nm) initiated photodynamic/chemodynamic therapy. This sequential laser‐induced activation enabled high‐performance NIRF image‐guided phototherapy for rectal tumours (Figure 2). Notably, delivering PSs to the deep tumour region led to an unprecedented therapeutic outcome: a single dose of P‐ZWNIR NPs with dual‐laser treatment on day 0 successfully eradicated tumours (∼250 mm^3^) while minimising the risk of recurrence.

P‐ZWNIR NPs challenge the traditional role of PDT as merely an adjunctive treatment. By integrating imaging and therapeutic modalities, these NPs demonstrated the potential to serve as a standalone treatment modality. This breakthrough holds transformative potential for tumours that elude surgical intervention, resist radiation therapy or present high‐risk treatment scenarios due to their critical anatomical locations. The multifunctionality of P‐ZWNIR NPs not only enhances treatment precision but also streamlines the diagnostic and therapeutic processes, offering a holistic approach to cancer management.

## 3 TOWARDS CLINICAL TRANSLATION: PERSPECTIVES AND FUTURE DIRECTIONS

While the preclinical success of P‐ZWNIR NPs is promising, translating this innovation into clinical practice requires addressing several key aspects. First, comparative studies are needed to benchmark P‐ZWNIR NP‐assisted therapies against conventional treatments, such as surgical resection and radiation therapy. The studies should evaluate parameters, including tumour control, local recurrence, distant metastasis, and both local and systemic side effects. Second, the shallow tissue penetration depth of 660 nm laser could pose a challenge for treating large solid tumours in humans.^4^ Advanced methods, such as two‐photon excitation, could be applied to enhance light penetration into deeper tissues. Third, tailored devices, including frontal diffuser fibres, cylindrical diffusing fibres and balloon catheters, are required to adapt PDT to a diverse array of tumour types and challenging anatomical locations encountered in clinical settings.^5^

P‐ZWNIR NPs represent a transformative advancement in PDT by overcoming critical limitations inherent to both PDT and NP‐based delivery systems in oncology. By seamlessly integrating imaging capabilities with enhanced tumour penetration and synergistic therapeutic effects, P‐ZWNIR NPs present a promising alternative to invasive treatments for addressing complex and challenging tumours. With ongoing research aimed at refining their design and expanding clinical applicability, P‐ZWNIR NPs are poised to become a foundational tool in the management of solid tumours.

## AUTHOR CONTRIBUTIONS

All authors contributed to the conception, writing, and review of the article.

## CONFLICT OF INTEREST STATEMENT

The authors declare they have no conflicts of interest.

## ETHICS STATEMENT

Not applicable.
